# Flowing through the CRISPR-CAScade: Will genome editing boost cell therapies?

**DOI:** 10.1186/2052-8426-1-3

**Published:** 2013-11-06

**Authors:** Uri Ben-David

**Affiliations:** Stem Cell Unit, Department of Genetics, Silberman Institute of Life Sciences, The Hebrew University, Jerusalem, 91904 Israel

**Keywords:** Genome editing, Cell therapy, Stem cells

## Abstract

Recent years have seen great advancements in genome editing technologies, allowing for efficient and specific targeting of DNA sequences into the genome. In parallel, advancements in stem cell research, and especially the ability to induce pluripotency in somatic cells, have brought stem cell-derived therapies closer to the clinic. In this commentary, I envision how groundbreaking genome editing technologies will influence stem cell biology research, paving the way to regenerative medicine with genetically engineered cells.

## Background

The ability to edit the genome of human stem cells in an efficient and site-specific manner is essential for the development of stem cell-based gene therapies. The rapid advancements in genome engineering technologies have thus arose much interest in the cell therapy field [1, 2]. Zinc-finger nucleases (ZFNs) [3] and transcription activator-like effector nucleases (TALENs) [4] fuse a DNA-binding domain to a DNA cleavage domain to create double strand breaks (DSBs) in specific genomic sequences. Both methods have been successfully applied to genome engineering in human pluripotent stem cells (hPSCs) [5, 6]. However, despite their seminal contribution to the genomic editing of human cells, the application of these methods remains relatively laborious and time consuming, as they require the engineering of specific restriction enzymes for each desired target.

## Discussion

Recently, a flow of studies has reported successful genome editing of mammalian cells using the CRISPR-Cas system [7–10]. The clustered, regularly interspaced, short palindromic repeats (CRISPR) system is a component of an immunity system of prokaryotes, both bacteria and archaea. The CRISPR-associated (Cas) endonuclease is directed by small RNAs to cleave foreign sequences of nucleic acids that penetrate the prokaryotic cell (reviewed in [11, 12]). Multiple groups have now shown that the CRISPR-Cas system can be manipulated to direct cleavage of desired target sequences in mammalian cells [7–10, 13, 14]. Applying this genome editing tool, mutations could be induced into specific genes following DSB induction and non-homologous end joining; most importantly, donor sequences could also be introduced by homologous recombination, demonstrating the practicability of this method for gene correction. Detailed explanations and illustrations of the CRISPR-Cas technology can be found in [7, 8].

In contrast to ZFNs and TALENs, CRISPR-Cas based targeting requires only the design of a new RNA guide sequence and not of new enzymes, making it much easier and cheaper. In addition, the CRISPR-Cas method is remarkably efficient, and several groups have already applied it successfully to mouse and human pluripotent stem cells [9, 13, 14]. Despite the understandable excitement, however, caution is warranted; as with any new technology, there are remaining challenges that have to be addressed before CRISPR-Cas becomes the gold standard of genome editing. One concern is the potentially high frequency of off-target mutagenesis induced by the CRISPR-Cas system in human cells [15, 16]. Another constraint is that the 20-bp target sequence must be followed by a protospacer adjacent motif (PAM), which might be a barrier for mutation correction at a specific genomic location [7, 8, 16]. Therefore, CRISPR should not be regarded as a revolutionizing technology that turns all previous methods obsolete; rather, it is an important promising step in the extraordinarily rapid evolution of genome editing techniques.

## Conclusions

**Figure 1:**
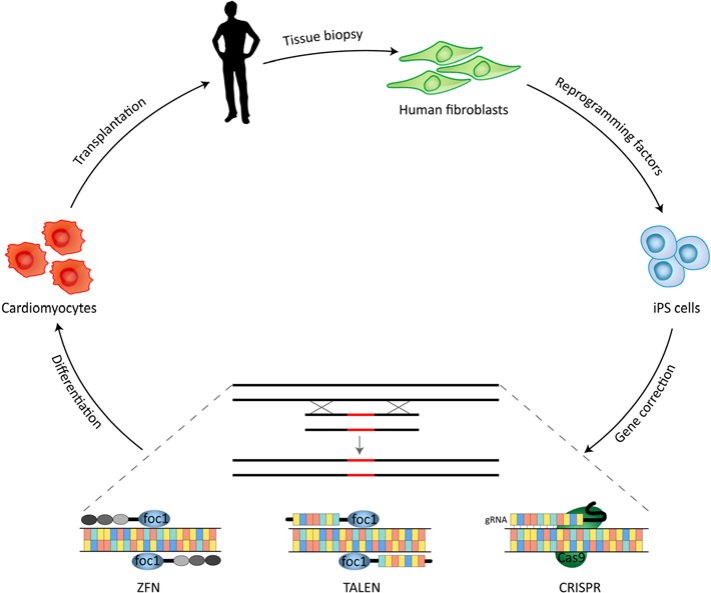
**Advancements in genome editing facilitate stem cell-based gene therapies.** In a probable scenario, human somatic cells (such as fibroblasts) will be derived from a patient and will be reprogrammed into induced pluripotent stem (iPS) cells. Genetic mutations will then be corrected using one of the recent techniques for efficient and accurate genome editing: ZFN, TALEN or CRISPR. The genetically-modified iPS cells will next be differentiated into the desired cell type (for example, cardiomyocytes) and transplanted back into the patient's body.

The cascade of already-published studies prompted by the original reports (just a few months ago!) of CRISPR-Cas based genome editing in mammalian cells, suggests that genome editing will soon become a routine procedure in many stem cell laboratories. An especially promising outcome of that would be the much-desirable possibility to perform genome editing with stem cells. Stem cell scientists will now be able to easily manipulate stem cells' genomes, inserting or correcting multiple genetic mutations, and then differentiate these stem cells into relevant cell types. Future studies will most likely attempt to integrate this novel technology into modeling various genetic disorders, and to examine its safety in preclinical and clinical trials. On top of novel insights into genetic diseases, which this approach is predicted to yield, it will also bring us one step closer to one of the most ambitious goals of regenerative medicine: combined gene- and cell-therapies, i.e. regenerative medicine with genetically-modified cells (see Figure 1).
